# The associations of body mass index with physical and mental aspects of health-related quality of life in Chinese patients with type 2 diabetes mellitus: results from a cross-sectional survey

**DOI:** 10.1186/1477-7525-11-142

**Published:** 2013-08-21

**Authors:** Carlos K H Wong, Yvonne Y C Lo, Winnie H T Wong, Colman S C Fung

**Affiliations:** 1 Department of Family Medicine and Primary Care, The University of Hong Kong, 3/F, Ap Lei Chau Clinic, 161 Ap Lei Chau Main Street, Ap Lei Chau, Hong Kong

**Keywords:** Quality of life, SF-12, Hong Kong, Chinese, Type 2 diabetes mellitus

## Abstract

**Background:**

This study aimed to determine the associations of various clinical factors with generic health-related quality of life (HRQOL) scores among Hong Kong Chinese patients with type 2 diabetes mellitus (T2DM) in the outpatient primary care setting using the short-form 12 (SF-12).

**Methods:**

A cross-sectional survey of 488 Chinese adults with T2DM recruited from a primary care outpatient clinic was conducted from May to August 2008. Data on the standard Chinese (HK) SF-12 Health Survey and patients’ socio-demographics were collected from face-to-face interviews. Glycaemic control, body mass index (BMI), chronic co-morbidities, diabetic complications and treatment modalities were determined for each patient through medical records. Associations of socio-demographic and clinical factors with physical component summary (PCS-12) and mental component summary scores (MCS-12) were evaluated using multiple linear regression.

**Results:**

The socio-demographic correlates of PCS-12 and MCS-12 were age, gender and BMI. After adjustment for socio-demographic variables, the BMI was negatively associated with PCS-12 but positively associated with MCS-12. The presence of diabetic complications was associated with lower PCS-12 (regression coefficient:-3.0 points, p < 0.05) while being on insulin treatment was associated with lower MCS-12 (regression coefficient:-5.8 points, p < 0.05). In contrast, glycaemic control, duration of T2DM and treatment with oral hypoglycaemic drugs were not significantly associated with PCS-12 or MCS-12.

**Conclusions:**

Among T2DM subjects in the primary care setting, impairments in the physical aspect of HRQOL were evident in subjects who were obese or had diabetic complications whereas defects in the mental aspect of HRQOL were observed in patients with lower BMI or receiving insulin injections.

## Background

The morbidity and mortality related to Type 2 diabetes mellitus (T2DM) have been increasing worldwide over past decades, particularly in Asia [1]. In Hong Kong, T2DM is the second leading type of chronic disease, accounting for 20% of chronic patients, with considerable prevalence of 15% in individuals aged 60–80 years [2] and 9.8% in the overall population [3].

Health-related quality of life (HRQOL) is defined as a multidimensional construct reflecting patients’ subjective perceptions of their physical, mental and social functioning [4]. Meanwhile, glycaemic control describes the presence of glucose in the blood, which is regarded as a critical outcome of diabetes care. Failure to achieve glycaemic control in diabetes was associated with the risks for developing diabetic complications such as cardiovascular diseases, stroke, blindness, leg amputation, kidney failure and renal failure, and may subsequently impair the HRQOL of T2DM patients. The impact of glycaemic control on HRQOL was examined by previous studies in which poor glycaemic control usually resulted in lower HRQOL as measured by diabetes-specific and generic health preference scores [5,6]. Besides, a higher body mass index (BMI) or clinical obesity (BMI ≥ 30 kg/m^2^) was mainly associated with a lower HRQOL [7-11]. Other factors that potentially affect patients’ HRQOL include duration of T2DM, use of oral hypoglycaemic drugs and insulin injections. There is little known in Chinese patients with T2DM receiving care in the primary care setting regarding the impact of these clinical factors on HRQOL, as available data are limited to the hospital-based specialist outpatient clinic setting [12,13].

Knowledge of the short-form 12 (SF-12) data is useful for comparing the impact of T2DM with those of other diseases and facilitating medical decision making. Nevertheless, data on the SF-12 among Hong Kong Chinese patients with T2DM have not been reported. Hence, the aim of this study is to report the distribution of SF-12 scores among Hong Kong Chinese patients with T2DM and to explore the factors associated with these scores.

## Methods

### Study design

A cross-sectional interviewer-administered survey was carried out at a government-funded general outpatient clinic at Ap Lei Chau in Hong Kong China from May to August 2008. Within the aforementioned period, there were 1,394 patients consulting at Ap Lei Chau clinic for T2DM. A convenience sampling of T2DM patients who attended the clinic was retrieved from electronic medical records. Selected patients were contacted prior to their appointment dates, informed about the study and invited to participate. On the day of their clinic visits, patients were recruited through referrals from clinicians, and screened according to the inclusion and exclusion criteria. The inclusion criteria were 1) diagnosis of non-insulin dependent diabetes mellitus with an International Classification of Primary Care-2 (ICPC-2) code of T90 and 2) written informed consent for participation in the study. The exclusion criteria were 1) inability to communicate in Cantonese or Chinese, 2) inability to carry out activities of daily living, 3) impaired cognitive function as assessed by recruiter, and 4) less than one year of life expectancy due to other co-morbidities as documented in the medical records. Data on patients’ socio-demographics and SF-12 were collected by a trained interviewer while clinical data were retrieved from electronic medical records. The study was approved by the Institutional Review Board of the University of Hong Kong/Hospital Authority Hong Kong West Cluster (HKU/HA HKW #UW 07–399).

### Outcome measures

#### Health-related quality of life

The SF-12 Health Survey is a shorter version of the 36-item SF-36 Health Survey. It comprises 12 items that measure physical functioning (PF), role physical (RP), role emotional (RE), bodily pain (BP), general health (GH), vitality (VT), social functioning (SF) and mental health (MH). The eight domain scores were aggregated into the physical and mental component summary scores (PCS-12 and MCS-12). The summary scores of PCS-12 and MCS-12, which were derived by the weighted sum of 12 items scores using the US standard SF-12 scoring algorithm [14], were considered as the primary outcomes of this study. The SF-12 has been shown to be not only valid and reliable but also equivalent to SF-36 in the Hong Kong population [14]. The first version of SF-12 was used in this study. No missing data for individual items were allowed to calculate the PCS-12 and MCS-12.

#### Clinical outcomes and socio-demographic information

We identified a few clinical variables that were reported to be associated with HRQOL among T2DM patients based on the literature [4,9,15]; these variables included glycaemic control, BMI, diabetic complications, hypertension, prescription of oral hypoglycaemic drugs and insulin regimen. Data on glycaemic control (HbA_1C_) and BMI were collected by clinical and laboratory assessments for each patient, whereas those on duration of T2DM, presence of T2DM-related complications (retinopathy, neuropathy, nephropathy, cardiovascular disease and other complications caused by diabetes), type of diabetes treatment, and presence of hypertension were extracted from medical records. In this study, retinopathy was defined as patients with potential loss of vision (background retinopathy, proliferative retinopathy or decreased vision); nephropathy was defined as patients having proteinuria or on dialysis; neuropathy was defined as having risk of foot ulceration, sepsis, amputation or Charcot joints; and cardiovascular disease was defined as having angina, previous myocardial infarction or congestive heart failure; the presence of hypertension was defined as patients with a history of hypertension in their medical record. The socio-demographic information including marital status, education level, monthly income and occupation was self-reported by patients. Marital status was classified as currently married or not currently married. Educational level was categorized as no formal, primary, secondary and tertiary schooling. Monthly income was classified as having less than the median of HKD$20,000 (pegged at an exchange rate of USD$1 = HKD$7.8), and having greater than or equal to HKD$20,000. Occupation was determined through British Registrar General’s classification and was classified into two categories: professional and skilled workers, and unskilled workers and others.

### Statistical analyses

Characteristics of the study samples are described using mean and standard deviation or proportions, where appropriate. As the associations of socio-demographic variables were first evaluated followed by clinical variables, hierarchical multiple linear regression was performed with PCS-12 and MCS-12 as outcome variables in separate models with and without the adjustment for other covariates: model 1 considered socio-demographic variables (age, gender, educational level, income, marital status, and occupation); model 2 adjusted for variables in model 1 and for the clinical variables (HbA_1C_ level, BMI level, duration of T2DM, treatment with oral hypoglycaemic drugs, treatment with insulin, presence of diabetic complications, and presence of hypertension). In model 3, a BMI-squared term was added to test for a potential non-linear relationship of BMI with MCS-12 and PCS-12. The clinical and demographic variables for adjustment were selected based on the conceptual framework developed by Rubin and Peyrot [4]. Duration of T2DM was categorized into two groups (≤ 5 years or > 5 years). Diabetes complications and hypertension were coded as present/absent. Treatments with oral hypoglycaemic drugs were coded as yes/no and likewise for treatment with insulin. Results from all regression models were presented with goodness-of-fit statistics in which R^2^ and adjusted R^2^ indicate the variance of PCS-12 and MCS-12 explained by the variables. It was hypothesized that PCS-12 and MCS-12 would be higher in patients with good glycaemic control, lower BMI, shorter disease duration, absence of DM complications or comorbidities, and being on oral rather than insulin treatment. The overall PCS-12 and MCS-12 scores in the current study were compared to those scores reported in a previous population-based survey [16], using independent *t*-test.

Patients with incomplete data on any of the variables used in multiple linear regressions were excluded from the analyses. All data analyses were performed in the SPSS Programme for Window 20.0 (IBM SPSS Inc. Chicago, IL, USA). P-values < 0.05 were defined as statistically significant.

## Results

The characteristics of study participants are summarized in Table 1. Mean age was 65.3 (SD: 11.0) years. Majority were female (62.1%), did not have formal education (40.6%), were married (72.7%), were semi-skilled workers (29.3%), and had a monthly income less than HKD$20,000 (70.7%).

**Table 1:** Characteristics of study participants (n = 488)

	**Duration of T2DM**			
	**Total**	**≤5 years (n = 265)**	**>5 years (n = 223)**	**P-value**
Age (years, mean ± SD)	65.34 ± 11.03	64.02 ± 11.63	66.90 ± 10.08	0.004
≤54 (%)	17.0%	20.80%	12.60%	<0.001
55–64 (%)	31.8%	36.2%	26.5%	
≥65 (%)	51.2%	43.0%	61.0%	
Gender				0.221
Male (%)	37.9%	40.4%	35.0%	
Female (%)	62.1%	59.6%	65.0%	
Educational Level				0.698
No Formal Education (%)	40.6%	40.0%	41.3%	
Primary School (%)	33.4%	32.5%	34.5%	
Secondary or above (%)	26.0%	27.5%	24.2%	
Marital Status				0.768
Married (%)	72.7%	72.3%	73.5%	
Non-Married* (%)	27.3%	27.7%	26.5%	
Occupation				0.157
Professionals & Associate Professionals (%)	3.1%	7.9%	13.5%	
Skilled Workers (%)	23.0%	24.5%	21.1%	
Semi-skilled Workers (%)	29.3%	28.7%	30.0%	
Unskilled Workers (%)	21.1%	23.8%	17.9%	
Others (%)	16.2%	15.1%	17.5%	
Monthly Income (HKD)				0.627
<$20,000 (%)	70.7%	88.3%	89.7%	
≥$20,000 (%)	11.1%	11.7%	10.3%	
HbA_1C_ (%, mean ± SD)	7.38 ± 1.27	7.20 ± 1.21	7.59 ± 1.30	<0.001
BMI (kg/m^2^, mean ± SD)	25.79 ± 4.00	26.15 ± 4.15	25.36 ± 3.77	0.030
Drug Treatment				
No Oral Hypoglycaemic Drugs (%)	23.0%	26.8%	18.4%	0.028
Oral Hypoglycaemic Drugs (%)	77.0%	73.2%	81.6%	
No Insulin Injections (%)	97.7%	99.6%	95.5%	0.002
Insulin Injections (%)	2.3%	0.4%	4.5%	
Any Diabetic Complications				0.430
Absent (%)	90.0%	90.9%	88.8%	
Present (%)	10.0%	9.1%	11.2%	
Retinopathy	3.1%	2.3%	4.0%	0.259
Neuropathy	0.8%	0.8%	0.9%	0.862
Nephropathy	2.0%	0.8%	3.6%	0.028
Cardiovascular Disease	4.5%	5.3%	3.6%	0.369
Others	0.2%	0.0%	0.4%	NA
Presence of Hypertension				0.083
Absent (%)	11.9%	24.9%	18.4%	
Present (%)	78.1%	75.1%	81.6%	
SF-12 Health Survey				
PCS-12 (mean ± SD)	42.45 ± 9.78	42.96 ± 9.60	41.84 ± 9.97	0.210
MCS-12 (mean ± SD)	51.64 ± 6.37	51.70 ± 6.29	51.57 ± 6.48	0.822

BMI body mass index, HbA1c glycated haemoglobin, MCS-12 Chinese (HK) SF-12 mental component summary, PCS-12 Chinese (HK) SF-12 physical component summary, SF-12 short-form 12, T2DM type 2 diabetes mellitus.

* Non-married includes single, widowed, separated, divorced and refusal.

The mean of HbA_1c_ levels of subjects was 7.4% (SD: 1.3), with 53.5% having HbA_1c_ > 7% and with more than 10% controlling T2DM badly (ie, with HbA_1c_ > 9%). The mean duration of diabetes for patients was 7.1 (SD: 6.3) years, and the mean BMI was 25.8 kg/m^2^and 53.01% had BMI ≥ 25 kg/m^2^. More than half of the patients (78.1%) had hypertension, and on the other hand, 90.0% of patients did not have any diabetes-related complication.

Descriptive statistics and clinical group comparisons PCS and MCS are presented in Table 2. Patients with chronic co-morbidities had less PCS-12 (42.00 ± 9.76 vs 46.52 ± 9.05) than patients without chronic co-morbidity. Presence of hypertension (44.99 ± 9.30 vs 41.74 ± 9.80) and diabetic complications (38.07 ± 11.01 vs 42.94 ± 9.52) were individually associated with poorer PCS-12. The PCS-12 was statistically lower in subjects treated with oral hypoglycaemic drugs (41.78 ± 9.77) than in subjects without oral medications (44.68 ± 9.52). Patients on insulin treatment had a significantly lower MCS-12 score, which further substantiates previous reports [12,17] of the adverse effect of insulin injections on HRQOL.

**Table 2:** Descriptive statistics of HRQOL scores, PCS-12 and MCS-12 by clinical characteristics

	**N = 488**	**PCS-12**		**MCS-12**	
		**Mean** ± **SD**	**P-value**	**Mean** ± **SD**	**P-value**
**Overall (N = 488)**		**42.45 ± 9.78**		**51.64 ± 6.37**	
HbA1C (%, mean ± SD)	7.38 ± 1.27				
Below Glycaemic Goal, ≤7.0 (%)	46.46%	41.89 ± 9.94	0.222	51.56 ± 6.20	0.793
Above Glycaemic Goal, >7.0 (%)	53.54%	42.99 ± 9.69		51.72 ± 6.46	
BMI (kg/m^2^, mean ± SD)	25.79 ± 4.00				
Underweight/normal, <23 (%)	22.87%	43.46 ± 10.18	0.196	50.53 ± 7.36	0.110
Overweight, 23 to <25 (%)	24.12%	43.17 ± 9.41		52.15 ± 6.00	
Obese, ≥25 (%)	53.01%	41.71 ± 9.75		51.89 ± 6.10	
Duration of T2DM (years, mean ± SD)	7.10 ± 6.27				
Duration ≤ 5 years (%)	54.30%	42.96 ± 9.60	0.210	51.70 ± 6.29	0.822
Duration > 5 years (%)	45.70%	41.84 ± 9.97		51.57 ± 6.48	
Lifestyle					
No Diet (%)	18.03%	43.01 ± 9.94	0.550	51.62 ± 6.54	0.978
Diet (%)	81.97%	42.32 ± 9.75		51.65 ± 6.34	
No Exercise (%) *	23.16%	40.48 ± 9.26	0.015	50.97 ± 6.74	0.200
Exercise (%) *	76.84%	43.04 ± 9.86		51.84 ± 6.25	
Drug Treatment					
No Oral Hypoglycaemic Drugs (%) *	22.95%	44.68 ± 9.52	0.006	51.10 ± 6.69	0.307
Oral Hypoglycaemic Drugs (%) *	77.05%	41.78 ± 9.77		51.80 ± 6.27	
No Insulin Injections (%) †	97.75%	42.54 ± 9.69	0.182	51.76 ± 6.25	0.007
Insulin Injections (%) †	2.25%	38.55 ± 12.95		46.57 ± 9.57	
Any Diabetic Complications					
Absent (%) *	89.96%	42.94 ± 9.52	0.001	51.63 ± 6.33	0.898
Present (%) *	10.04%	38.07 ± 11.01		51.75 ± 6.80	
Any Chronic Co-morbidities					
Absent (%) *	9.84%	46.52 ± 9.05	0.002	50.67 ± 7.09	0.268
Present (%) *	90.16%	42.00 ± 9.76		51.75 ± 6.29	
If present, how many (mean ± SD)	1.87 ± 0.98				

Note:*BMI* body mass index, *HbA*_*1c*_ glycated haemoglobin, *MCS-12* Chinese (HK) SF-12 mental component summary, *PCS-12* Chinese (HK) SF-12 physical component summary, *SF-12* short-form 12, *T2DM* type 2 diabetes mellitus

* Significant difference (p < 0.05) on PCS-12 between groups by independent samples *t*-test.

† Significant difference (p < 0.05) on MCS-12 between groups by independent samples *t*-test.

Table 3 shows the results from multiple linear regression analyses of HRQOL associated clinical factors, before (model 1) and after adjustment (model 2) for socio-demographic factors. Among all the socio-demographic factors studied, age and gender were significantly associated with both measures of PCS-12 and MCS-12 in all regression analyses. Based on model 2 which had adjusted for socio-demographic factors, the association of BMI with HRQOL was different in terms of PCS-12 and MCS-12. Significant, positive association was found between BMI and MCS whereas negative association was observed for PCS. Patients on insulin treatment reported significantly worse MCS-12 with more than 5.8 points decline. The presence of DM complications imposed a substantial negative impact on PCS-12, while hypertension was not significantly associated with HRQOL. HbA_1C_ level, duration of T2DM and treatment with oral hypoglycaemic drugs were not significantly associated with PCS-12 or MCS-12. No evidence of a non-linear effect of the BMI was found, as the BMI-squared term was not significant and small changes of adjusted R^2^ (PCS-12: -0.001; MCS-12: 0.010) from model 2 to model 3 were reported for PCS-12 and MCS-12 analysis when it was added to model 3.

**Table 3:** Clinical and socio-demographic factors associated with PCS-12 and MCS-12 by Hierarchical Multiple Linear Regression

	**PCS-12**				**MCS-12**		
	**Coeff.**	**95% CI**	**P-value**		**Coeff.**	**95% CI**	**P-value**
**Model 1 (R**^**2**^ **= 0.216; Adj R**^**2**^ **= 0.206)**				**Model 1 (R**^**2**^ **= 0.035; Adj R**^**2**^ **= 0.023)**			
**Socio-demographic**				**Socio-demographic**			
Age	−0.29	(−0.37,−0.20)	<0.001*	Age	0.07	(0.01,0.13)	0.020*
Female	−3.50	(−5.39,−1.60)	<0.001*	Female	−1.60	(−2.96,−0.24)	0.021*
Educational (No formal education)				Educational (No formal education)			
Secondary or above	1.76	(−0.61,4.13)	0.145	Secondary or above	−0.89	(−2.59,0.81)	0.304
Primary School	−0.91	(−2.93,1.10)	0.374	Primary School	−2.04	(−3.49,−0.59)	0.006*
Married	1.56	(3.49,−0.37)	0.114	Married	0.09	(1.48,−1.29)	0.894
Professional and Skilled	1.11	(−0.79,3.02)	0.253	Professional and Skilled	0.47	(−0.90,1.83)	0.505
Monthly Income ≥ HKD$20,000	1.14	(−1.51,3.79)	0.399	Monthly Income ≥ HKD$20,000	0.93	(−0.97,2.83)	0.338
**Model 2 (R**^**2**^ **= 0.252; Adj R**^**2**^ **= 0.229)**				**Model 2 (R**^**2**^ **= 0.074; Adj R**^**2**^ **= 0.045)**			
**Socio-demographic**				**Socio-demographic**			
Age	−0.30	(−0.38,−0.21)	<0.001*	Age	0.09	(0.03,0.15)	0.005*
Female	−3.48	(−5.36,−1.60)	<0.001*	Female	−1.73	(−3.08,−0.39)	0.012*
Educational (No formal education)				Educational (No formal education)			
Secondary or above	1.11	(−1.25,3.47)	0.358	Secondary or above	−0.54	(−2.24,1.15)	0.530
Primary School	−1.28	(−3.30,0.73)	0.211	Primary School	−1.79	(−3.23,−0.35)	0.015*
Married	1.46	(−0.44,3.36)	0.133	Married	0.23	(−1.13,1.60)	0.736
Professional and Skilled	1.09	(−0.80,2.97)	0.259	Professional and Skilled	0.63	(−0.72,1.99)	0.361
Monthly Income ≥ HKD$20,000	1.11	(−1.50,3.72)	0.405	Monthly Income ≥ HKD$20,000	0.92	(−0.95,2.79)	0.333
**Clinical**				**Clinical**			
HbA1C	0.05	(−0.58,0.68)	0.872	HbA1C	0.06	(−0.39,0.51)	0.801
BMI	−0.28	(−0.49,−0.08)	0.006*	BMI	0.15	(0.00,0.29)	0.048*
Duration of DM >5 years	−0.25	(−1.86,1.36)	0.763	Duration of DM >5 years	−0.05	(−1.21,1.10)	0.931
Oral Hypoglycaemic Drug	0.56	(−2.51,3.64)	0.719	Oral Hypoglycaemic Drug	−1.09	(−3.29,1.12)	0.334
Insulin Injection	−3.79	(−9.02,1.45)	0.156	Insulin Injection	−5.80	(−9.56,−2.05)	0.002*
DM complications	−3.04	(−5.66,−0.42)	0.023*	DM complications	0.31	(−1.57,2.19)	0.743
Hypertension	−1.55	(−4.67,1.57)	0.330	Hypertension	1.99	(−0.25,4.23)	0.082
**Model 3 (R**^**2**^ **= 0.252; Adj R**^**2**^ **= 0.228)**				**Model 3 (R**^**2**^ **= 0.085; Adj R**^**2**^ **= 0.055)**			
**Socio-demographic**				**Socio-demographic**			
Age	−0.30	(−0.38,−0.21)	<0.001*	Age	0.09	(0.03,0.15)	0.005*
Female	−3.56	(−5.45,−1.66)	<0.001*	Female	−1.81	(−3.17,−0.45)	0.009*
Educational (No formal education)				Educational (No formal education)			
Secondary or above	1.10	(−1.26,3.46)	0.362	Secondary or above	−0.55	(−2.25,1.14)	0.521
Primary School	−1.31	(−3.33,0.70)	0.201	Primary School	−1.82	(−3.26,−0.38)	0.013*
Married	1.43	(−0.47,3.34)	0.140	Married	0.21	(−1.16,1.57)	0.765
Professional and Skilled	1.04	(−0.85,2.94)	0.279	Professional and Skilled	0.59	(−0.77,1.94)	0.396
Monthly Income ≥ HKD$20,000	1.14	(−1.47,3.74)	0.393	Monthly Income ≥ HKD$20,000	0.95	(−0.92,2.82)	0.318
**Clinical**				**Clinical**			
HbA1C	0.06	(−0.57,0.68)	0.863	HbA1C	0.06	(−0.39,0.51)	0.788
BMI	−0.80	(−2.42,0.82)	0.334	BMI	−0.39	(−1.55,0.77)	0.508
BMI^2^	0.01	(−0.02,0.04)	0.529	BMI^2^	0.01	(−0.01,0.03)	0.358
Duration of DM >5 years	−0.24	(−1.85,1.37)	0.767	Duration of DM >5 years	−0.05	(−1.20,1.11)	0.937
Oral Hypoglycaemic Drugs	0.59	(−2.48,3.66)	0.706	Oral Hypoglycaemic Drugs	−1.06	(−3.26,1.14)	0.346
Insulin Injections	−3.82	(−9.05,1.42)	0.153	Insulin Injections	−5.83	(−9.59,−2.08)	0.002*
DM complications	−3.04	(−5.66,−0.42)	0.023*	DM complications	0.31	(−1.56,2.19)	0.743
Hypertension	−1.46	(−4.59,1.67)	0.361	Hypertension	2.08	(−0.16,4.33)	0.069

Note: BMI body mass index, HbA1c glycated haemoglobin, HT Hypertension, MCS-12 Chinese (HK) SF-12 mental component summary, PCS-12 Chinese (HK) SF-12 physical component summary, SF-12 short-form 12, T2DM type 2 diabetes mellitus.

*Significant difference (p < 0.05) on regression coefficient.

Table 4 compares the PCS-12 and MCS-12 results between the study and general population. The mean PCS-12 score of the study participants was significantly lower than that of the general population (42.45 ± 9.78 vs 49.99 ± 9.23, p < 0.05). The mean MCS-12 scores were comparable between the two populations (51.64 ± 6.37 vs 50.11 ± 9.53).

**Table 4:** Comparison of PCS-12 and MCS-12 results from Chinese Hong Kong Population

**SF-12 Summary Scores**		**Present study (n = 488)**	**2008-2009 HK general population study (n = 2,533)**
PCS-12* (mean ± SD)		42.45 ± 9.78	49.99 ± 9.23
MSC-12 (mean ± SD)		51.64 ± 6.37	50.11 ± 9.53

Note: *MCS-12* Chinese (HK) SF-12 mental component summary, *PCS-12* Chinese (HK) SF-12 physical component summary, *SF-12* short-form 12.

*Significant difference (p < 0.05) between studies by independent samples *t*-test.

## Discussion

Here we reported the first study that demonstrated the value of generic instrument SF-12 in assessing HRQOL in Chinese T2DM patients and examined the associated clinical and socio-demographic factors. Generic HRQOL instrument like SF-12 is sensitive to detect significant differences in HRQOL scores between different socio-demographic factors of age and gender in Hong Kong general population [16,18]. With a focus on T2DM patients, our study confirmed that age and gender were associated with both PCS-12 and MCS-12. Specifically, age was negatively associated with PCS-12 but positively with MCS-12, whereas female gender was negatively associated with both measures. Similar patterns of the socio-demographic factors on HRQOL were captured by a survey with reference to Chinese professional drivers in Hong Kong [13].

BMI was the only significant clinical predictor of both PCS-12 and MCS-12 in our multivariate analyses, after accounting for socio-demographic and clinical factors. Previous findings showed that obesity impaired physical health status and lower BMI was associated with higher PCS-12 [10]. According to a review by Kushner et al. [19], more severe types of obesity should in general have negative impacts on both physical and mental aspects of HRQOL, which was opposite to our result that lower BMI levels were consistently associated with lower MCS-12, but not with lower PCS-12. These completely different effects of BMI on PCS-12 and MCS-12 were also reported in other studies [13], in line with the ‘jolly fat’ hypothesis [20] indicating obese subjects perceived better mental aspect of HRQOL. Even when the univariate analysis appeared to show a non-linear relationship of BMI with MCS-12, the multivariate analyses did not provide evidence for a non-linear relationship as the coefficient before BMI-squared term is not significant for MCS-12 or PCS-12 in addition to the small change in adjusted R^2^ between models with and without BMI-squared term.

This study demonstrated the contrary associations of BMI with physical and mental aspects of HRQOL, and urged caution on the statement that lower BMI in T2DM subjects was associated with better HRQOL regardless of physical and mental aspects. Our finding also points to an unmet need in patient education – a lack of self-discipline in diet and physical activity among overweight/obese patients may account for their seemingly better mental health and compromised physical health compared with patients with a lower BMI. In educating these patients, it is important for clinicians to highlight the negative impacts of obesity on physical health, in order to motivate patients to implement necessary lifestyle modifications. Building a social supportive network is a feasible approach to improving patients’ psychosocial health, and reinforcing their motivation to maintain a healthy lifestyle.

Diabetic complications and insulin injection treatment were two predictors of poor HRQOL identified in our study. Several studies showed [8,21-23] that the increase in the number of diabetic complications would lead to impairment of PCS-12 and generic health preference scores. There is little evidence that injection regimen would be beneficial to HRQOL of T2DM patients. Two UK studies found that compared with oral hypoglycaemic drug treatment alone, insulin injection treatment significantly improved patients’ subjective well-being [24], in particular, at the expense of a higher degree of anxiety [25]. Our study provided evidence on the deterioration of mental HRQOL upon injection regimen. Moreover, our study demonstrated that glycaemic control and duration of T2DM were not significantly associated with HRQOL in patients with T2DM, in contrast to the conventional perception that glycaemic control was an important determinant of quality of life as reported in previous studies [5,26,27]. Another study conducted in Hong Kong also reported a lack of association between HRQOL with glycaemic control and duration of T2DM, suggesting that this might be a culturally-specific finding [12]. Another reason was the adaption of generic instruments to measure HRQOL. Generic instruments provided more information on daily living and functioning related to quality of life rather than lifestyle and dietary issues [28-30] whereas diabetic-specific HRQOL instruments measured the quality of life impacted by diabetes. By convention, generic instruments were recommended for use in conjunction with diabetic-specific HRQOL instruments because diabetic-specific instruments would be more responsive to changes in quality of life scores between good and bad glycaemic control, compared with generic HRQOL instruments [4,28,31]. While disease-specific measures offer greater sensitivity and responsiveness compared with generic measures of HRQOL, generic measures allow for comparing the impact of diseases on HRQOL across various diseases and are more useful for guiding resource allocation. Both generic and disease-specific HRQOL measures would be used in tandem, with consideration of study objectives and patient burden [4].

There were several limitations for this study. First, caution is warranted that only association but not causality could be established by this cross-sectional study. Convenience sampling also limits the representativeness of the study sample. For example, insulin users who are frequently managed in secondary care may be under-represented in our study. Secondly, the health behavioural factors (e.g. smoking, drinking, diabetes self-management activities) and non-vascular diseases (e.g. musculoskeletal and emotional disorders) were not controlled for in the multivariate analyses. Regular physical exercise, as the highest positive indicator in the PCS-12 score, might be affected by those health behavioural factors. Further investigations are warranted to explore whether the associations of the clinical variables with HRQOL are medicated or partly mediated by these lifestyle factors. Thirdly, DM complications and chronic co-morbidities were recorded as numbers if present, without grading the severity of each complications and co-morbidities. Further studies on stratifying the severity of complications, e.g. into macrovascular and micro-vascular complications, are warranted, as suggested by previous studies [21,27,32]. Fourthly, the number of insulin users in this study was small (n = 11), as quality of life scores were not documented for insulin users with or without combinational treatment. Further studies are required to prove the impact of diabetic symptoms, behavioural and psychological factors on both generic and diabetic-related HRQOL instruments regarding Chinese patients with T2DM. Finally, the response rate of this study was not reported because the reasons for non-participation and number of subjects who were approached but declined to participate were not recorded in the data collection process.

## Conclusions

This study suggested that for patients with T2DM, obesity and diabetic complications affected their physical HRQOL, while insulin injections affected patients’ mental HRQOL, informing the deficiency of HRQOL as a barrier to optimal diabetes management and control in primary care settings in Hong Kong.

## Abbreviations

BMI: Body mass index; HbA1C: Glycated haemoglobin; HRQOL: Health-related quality of life; HT: Hypertension; MCS-12: Chinese (HK) SF-12 mental component summary score; PCS-12: Chinese (HK) SF-12 physical component summary score; SF-12: Chinese (Hong Kong) short-form 12; T2DM: Type 2 diabetes mellitus.

## Competing interests

The authors declare that they have no competing interests.

## Authors’ contributions

CW and YL provided direct input into the design and execution of the study. CW undertook statistical analysis and generated the results. CW, YL and CF drafted the manuscript and CW, YL, CF and WW contributed to its editing. All authors read and approved the final manuscript.
